# Adverse effects of ovarian cryopreservation and auto-transplantation on ovarian grafts and quality of produced oocytes in a mouse model

**DOI:** 10.1042/CS20230483

**Published:** 2023-10-18

**Authors:** Que Wu, Gaizhen Ru, Wanfen Xiao, Qian Wang, Zhiling Li

**Affiliations:** Reproductive Center, First Affiliated Hospital of Shantou University Medical College, Shantou University, Shantou City, 515041, Guangdong, China

**Keywords:** Cryopreservation, Hypoxia-ischaemia, Mitochondria function, Oocytes, Ovarian tissue, Transplantation

## Abstract

The process of ovarian cryopreservation and transplantation is the only feasible fertility preservation method for prepubertal girls and female patients with cancer who cannot delay radiotherapy and chemotherapy. However, basic research on this technique is lacking. To better understand ovarian function and oocyte quality after ovarian tissue (OT) transplantation, we characterised the appearance, angiogenesis, and endocrine function of ovarian grafts in a murine model; the mitochondrial function and DNA damage in oocytes isolated from the OT; and the development of embryos after *in vitro* fertilisation. The results showed a decrease in oocyte numbers in the transplanted OT, abnormal endocrine function of ovarian grafts, as well as dysfunctional mitochondria and DNA damage in the oocytes, which could adversely affect subsequent embryonic development. However, these adverse phenotypes were partially or completely resolved within 21 days of transplantation, suggesting that ovulation induction and assisted pregnancy treatment should not be conducted too soon after OT transfer to ensure optimal patient and offspring outcomes.

## Introduction

Over the past few decades, improvements in cancer diagnosis and treatment have dramatically increased the long-term survival rates of many young female patients with cancer. However, chemotherapy and radiotherapy can trigger early menopause, premature ovarian failure, and infertility in survivors [1]. As such, female fertility preservation is becoming increasingly important to improve patient quality of life and assist family planning [2]. While the cryopreservation of gametes and embryos is an established procedure for fertility preservation, it is not suitable for prepubertal children or women who cannot delay cancer therapy. However, ovarian tissue (OT) cryopreservation, with the advantage of not having to perform controlled hyperovulation, can preserve ovarian endocrine function after transplantation; thus, OT cryopreservation is currently the only feasible means for maintaining fertility in these patients [3,4].

Despite more than 200 live births resulting from OT cryopreservation and transplantation (OTCT) [5], the pregnancy and live birth rates remain poor compared with natural conception [6]. The poor outcomes of OTCT have been speculated as having two causes [7]: first, cryoinjury during the cryopreservation-warming protocol may cause stromal destruction and follicular loss [8]; second, and more serious, early ischaemia–reperfusion injury after transplantation [9] can cause follicular loss and reactive oxygen species (ROS) accumulation, leading to metabolic abnormalities and oxidative stress that lasts until the OT vasculature stabilises [7,9]. Previous studies have suggested that mouse graft revascularisation begins 2 days post-transplantation, and graft oxygen saturation is restored 7 days post-transplantation. However, OT requires approximately 21 days to achieve vasculature stabilisation [7,10–12].

With the objective of reducing cryoinjury and ischaemic damage, studies have investigated the use of an open freezing system [13] and supplementation with additives, such as antifreeze proteins [14], angiogenesis factors [15,16], and antioxidants [17–19]. These measures aimed to improve the ovarian transplantation success rate and surviving oocyte numbers. However, several questions remain unanswered:

1. What is the recovery status of ovarian function and neovascularisation on different days post-freezing?
2. Do oocytes growing in a hypoxic-ischaemic environment experience energy metabolism dysfunction affecting fertilisation potential?
3. What is the relationship between oocyte quality and OT recovery post-transplantation?
4. Do cryoinjury or ischaemic injury cause OT metabolic dysfunction post-transplantation, and is it time dependent?

To answer these questions, we transplanted OT with/without cryopreservation into mice and attempted to isolate cumulus-oocyte complexes from transplanted ovaries over a 3-week span following transplantation. In doing so, we focused on exploring the effects of OTCT on ovarian function and oocyte quantity and quality, through characterisation of the appearance, angiogenesis, and endocrine function of ovarian grafts on different days following ovarian transplantation. We further performed investigation of mitochondrial function, DNA damage, and developmental potential in oocytes isolated from the ovarian tissue.

## Methods

### Study design

**Figure 1:**
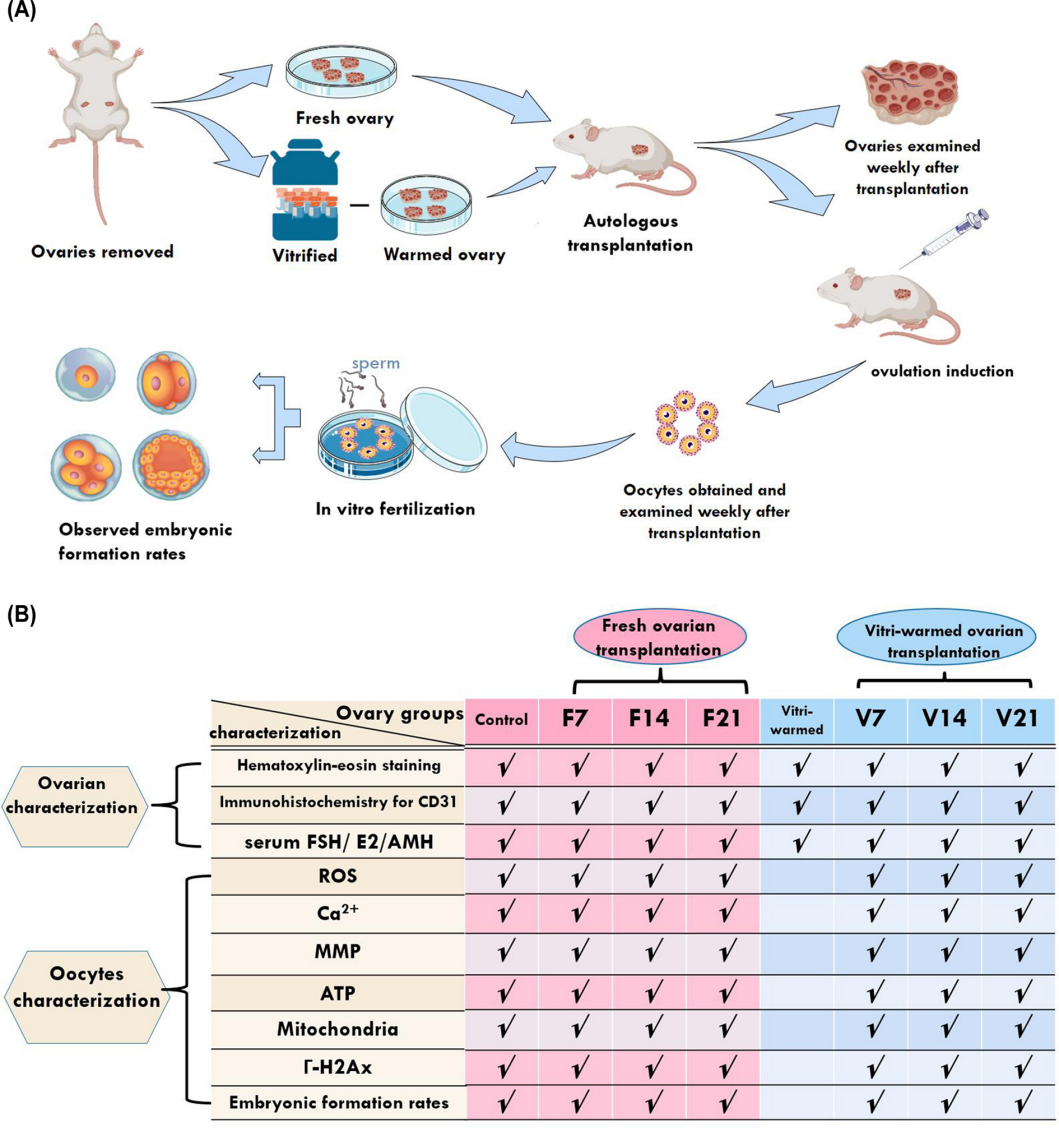
Experimental study design (**A**) Experimental procedure. (**B**) Ovarian and oocyte grouping and characterisation. **✓** indicates that this parameter was measured in this group. The fresh ovarian transplantation groups were designated as F7, F14, and F21, with collections on post-transplantation day 7, 14, and 21, respectively. The vitri-warmed ovarian groups were similarly named V7, V14, and V21. γ-H2AX, nuclear γ-H2A histone family member X; AMH, anti-Müllerian hormone; E2, estradiol; FSH, follicle-stimulating hormone; MMP, mitochondrial membrane potential; ROS, reactive oxygen species. *Figure 1a was Created with BioRender.com*.

We used a murine model to explore the effects of ischaemic injury and/or cryoinjury during OTCT on the quality of ovaries and the resulting follicles. Fresh ovaries collected from healthy, fertile female mice without any treatment served as the control group (*n*=34); the treatment group was divided into two parts: freshly transplanted ovaries (fresh group) and vitrification-warmed transplanted ovaries (vitri-warmed group). The treatment steps of the experimental groups are shown in Figure 1A. In the fresh group, autologous transplantation was performed immediately after the ovaries were removed. In the vitri-warmed group, the ovaries were vitrified in liquid nitrogen for one week and rewarmed prior to auto-transplantation. We characterised the ovaries weekly over a 3-week span following transplantation. Ovulation was induced in all other mice weekly over a 3-week span after transplantation. A series of tests for mitochondrial function, DNA damage, and the developmental potential of embryos after *in vitro* fertilisation were performed weekly on cumulus-oocyte complexes (COCs) isolated from transplanted ovaries over a 3-week span after transplantation. *In vitro* fertilisation was performed on these oocytes to observe the embryo formation rates. Ovarian and oocyte groups and characterisation parameters are shown in Figure 1B.

As the murine ovulation cycle occurs every 4 days, we set the detection cycle at 7 days to avoid collecting follicles in the same ovulation cycle. The fresh ovarian transplantation groups were designated as F7 (*n*=30), F14 (*n*=30), and F21 (*n*=32), with collections on post-transplantation day 7, 14, and 21, respectively. The vitri-warmed ovarian groups were similarly named V7 (*n*=29), V14 (*n*=30), and V21 (*n*=30). Ovaries and oocytes from each collection were characterised. For mouse ovaries (*n*=8) that underwent vitrification, cryopreservation, and rewarming without transplantation (vitri-warmed group), only ovarian characterisation was performed as no mature oocytes developed (Figure 1B).

Ovarian characterisation included haematoxylin-eosin (HE) staining, immunohistochemistry for CD31 as a count of microvessel density, and serum follicle-stimulating hormone (FSH), estradiol (E2), and anti-Müllerian hormone (AMH) concentrations. Oocyte characterisation included intracellular ROS and Ca^2+^ fluorescence, mitochondrial membrane potential (MMP), ATP-mitochondrial fluorescence double staining, nuclear γ-H2A histone family member X (γ-H2AX)-positivity, and embryo formation rates after *in vitro* fertilisation (IVF).

### Model animals

Adult Kunming female (3–6 weeks, *n*=223) and male (2–3 months, *n*=30) mice were obtained from the Animal Center of Shantou University Medical College. The present study was approved by the Experimental Animal Ethics Committee of our organisation (SUMC2022-593). All experimental protocols were performed in accordance with the International Guiding Principles for Biomedical Research Involving Animals (2012 version) issued by the Council for International Organizations of Medical Sciences. All mice were housed under standard conditions of a 12-h light/dark cycle, relative humidity (65% ± 5%), and temperature (24°C ± 2°C) and were fed standard rodent chow with ad libitum access to autoclaved water. Animal experiments were performed in the sterile animal operating room and Laboratory of Molecular Cardiology of Shantou University Medical College. All mice were killed by carbon dioxide asphyxiation in the experiment.

### OT collection, cryopreservation, warming, and transplantation

For anesthesia, female mice were injected intraperitoneally with Avertin (1.25% 2,2,2-tribromoethanol, Aibei, Nanjing, China) at 4–8 weeks of age. The OT was completely removed, washed in sterile saline, and cut into small pieces of approximately 1–2 mm^3^ after piercing the large follicles using a syringe needle. For the fresh group, the OT was immediately transplanted into an autologous renal capsule due to the robust renal blood supply [15], while OT for the vitrification group was treated with a surviving tissue cryopreservation and resuscitation kit (Huicun Medical Company, Shanghai, China). Following the kit instructions, the OT was placed at room temperature (RT) in V1 solution for 10 min, followed by V2 for 20 min, before being placed in liquid nitrogen. One week later, the OT was removed from liquid nitrogen, placed into T1 solution at 37°C for 3 min, and then placed in T2 and T3 solutions at RT for 10 min each. Subsequently, the warmed OT was transplanted into the autologous renal capsule following anaesthetisation of the mice. After completion of abdominal surgery, the peritoneum, muscle fascia, and skin of the mice were sutured with 5-0 sutures, and the mice were placed on a pet electric blanket to wake up.

### OT collection, HE staining, and immunohistochemistry for CD31

Ovaries were collected and immediately fixed in 4% paraformaldehyde. Paraffin sections (4 μm thick, every fifth section) were mounted on slides and stained with HE and subsequently observed under a microscope (Nikon Eclipse90 Ni-E; Tokyo, Japan) to determine the follicular number at 400× magnification.

CD31 immunostaining of OT was performed to detect microvessels as previously described [7]. Briefly, OT sections were treated with blocking solution and non-specific staining blockers (Maixin, Fujian, China) for 10 min sequentially after signal enhancement and cool down and subsequently incubated with anti-CD31 antibody (1:1000, Proteintech, Chicago, IL, U.S.A.) overnight at 4°C. The slides were incubated with a horseradish peroxidase-conjugated secondary antibody (Maixin, Fujian, China) for 10 min and then with DAB + substrate (Maixin, Fujian, China) for 3–5 min at RT. Slides were stained with haematoxylin and examined under a microscope (Nikon Eclipse90 Ni-E; Tokyo, Japan). After identifying the area with dense CD31 expression in the 200× field of view, three fields of view were selected at 400× magnification to count microvessels less than 20 μm in diameter.

### Serum FSH, E2, and AMH detection

Whole blood samples were collected via orbital venous bleeding, and serum was obtained after centrifugation. Blood samples from the control group were collected immediately after euthanasia. The serum samples obtained on the 7th day after ovariectomy (without transplantation) were set as the ovary removal group. All serum samples were analysed for FSH, E2, and AMH levels using chemiluminescence (Roche Cobas e601, Germany).

### Collection of oocytes and assessment of mitochondrial function

Superovulation was induced by consecutive intraperitoneal injections of 10 IU pregnant mare serum gonadotropin (PMSG) and 10 IU human chorionic gonadotropin (HCG) 48 h later [20]. From 11 to 12 h after HCG administration, COCs were obtained under an optical microscope by mechanical separation using two 1 ml syringes. Subsequently, 1 mg/mL hyaluronidase (Aibei, Nanjing, China) was used to remove the surrounding cumulus cells from the complexes to obtain oocytes.

Indicators of mitochondrial function were detected using fluorescence staining on these oocytes: 2′,7′-Dichlorodihydrofluorescein diacetate (10 μM, Sigma, MO, U.S.A.) for intracellular ROS detection, JC-1 (1.25 μM, MedChemExpress, New Jersey, U.S.A.) for MMP detection, and Fluo-4 AM (5 μM, Beyotime, Shanghai, China) for intracellular Ca^2+^ detection. Following a 30 min incubation at 37°C, all cells were examined immediately under a fluorescence microscope (EVOS M5000, Thermo Fisher Scientific, U.S.A.).

To understand the relationship between the mitochondria number and ATP production, we co-stained mitochondria and ATP using the fluorescence probes Mito-tracker Green (200 nM, Beyotime, Shanghai, China) and ATP-Red1 (5 μM, MedChemExpress, New Jersey, U.S.A.) in M2 medium. Oocytes were incubated at 37°C with the dye for 30 min. After treatment with an anti-fluorescence quencher (including Hoechst33342, Beyotime, Shanghai, China), oocytes were examined immediately under a fluorescence microscope. ImageJ (NIH Image, Bethesda, MD, U.S.A.) was used to quantify the fluorescence intensity.

### Indirect oocyte immunofluorescent staining for γH2AX

We used a DNA Damage Assay Kit with γ-H2AX immunofluorescence (Beyotime, Shanghai, China) to assess collected oocytes. Following the manufacturer’s instructions, we gradually fixed, blocked, and incubated the samples with primary and secondary antibodies; stained the nuclei of the oocytes; and counted the positive expression rate of γ-H2AX under a microscope (EVOS M5000, Thermo Fisher, U.S.A.).

### Oocyte IVF and embryo culture

All reagents were obtained from Aibei, Nanjing, China. COCs were collected in 37°C phosphate-buffered saline and incubated in *in vitro* maturation solution for 2 h. The oocytes were subsequently transferred to prepared 37°C fertilisation liquid, under oil and containing 10 μl of capacitated sperm collected from the male mouse epididymal tail and vas deferens. These were then incubated in human tubal fluid medium at 37°C for 4 h in a 5% CO_2_ incubator to permit fertilisation. Zygotes were washed three times and cultured in KSOM medium to observe embryonic development and to determine the 2-cell, 4-cell, and blastocyst formation rates.

### Statistical analysis

All tests were repeated at least thrice. One-way analysis of variance followed by a post-hoc test was performed to evaluate differences among means. Tukey’s test for multiple comparisons was used to compare each column’s mean with all other means. Analyses were performed using SPSS software (version 23.0; IBM, Armonk, NY, U.S.A.), and GraphPad Prism (version 9.0; GraphPad Software Inc, San Diego, CA, U.S.A.) was used to draw cartograms. Differences with *P*<0.05 were considered statistically significant.

## Results

### Examination of OT after auto-transplantation

**Figure 2:**
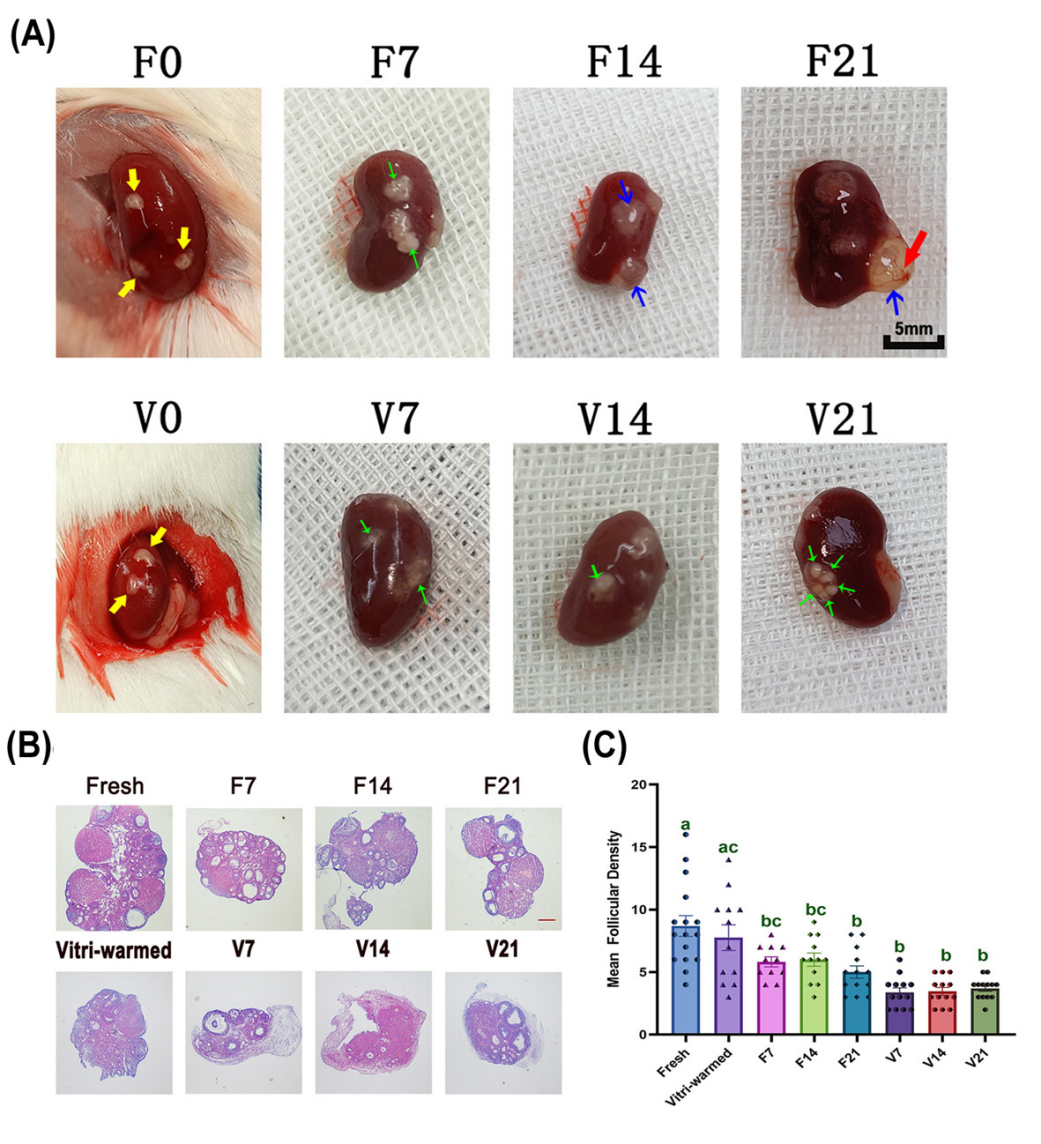
The OT after auto-transplantation (**A**) OT under the renal capsule after auto-transplantation. The yellow arrow indicates OT immediately transplanted into the renal capsule. The green, blue, and red arrows indicate mature follicles, accumulated mature follicular fluid, and new blood vessels, respectively; scale bar: 5 mm. (**B**) HE staining of OTs; scale bar: 50 μm. (**C**) The mean follicular density. Data are represented as means ± standard error. Individual data points are indicated by circles, triangles, and diamonds. The between group statistical significance (*P*<0.05) is indicated by lower case letters, such that the bars labelled with a certain letter are not statistically different from each other but are statistically different from the bars without that letter. The fresh ovarian transplantation groups were designated as F7, F14, and F21, with collections on post-transplantation day 7, 14, and 21, respectively. The vitri-warmed ovarian groups were similarly named V7, V14, and V21; OT, ovarian tissue.

Grafts from fresh and vitri-warmed ovaries both survived well under the renal capsule after autotransplantation (Figure 2A). Mature follicles, accumulated follicular fluid, and new small blood vessels were visible by post-transplantation day 7; however, the volume of the grafts in the fresh group was larger than that in the vitri-warmed group. HE staining of the OTs revealed several antral follicles on post-transplantation day 7 (Figure 2B). The number of follicles decreased post-transplantation (Figure 2C) in both fresh and vitri-warmed ovaries (*P*<0.05), and the decrease in the number of follicles in the vitri-warmed group was more obvious than that in the fresh group grafts.

### Reconstruction of microvessels and recovery of endocrine function in grafts

**Figure 3:**
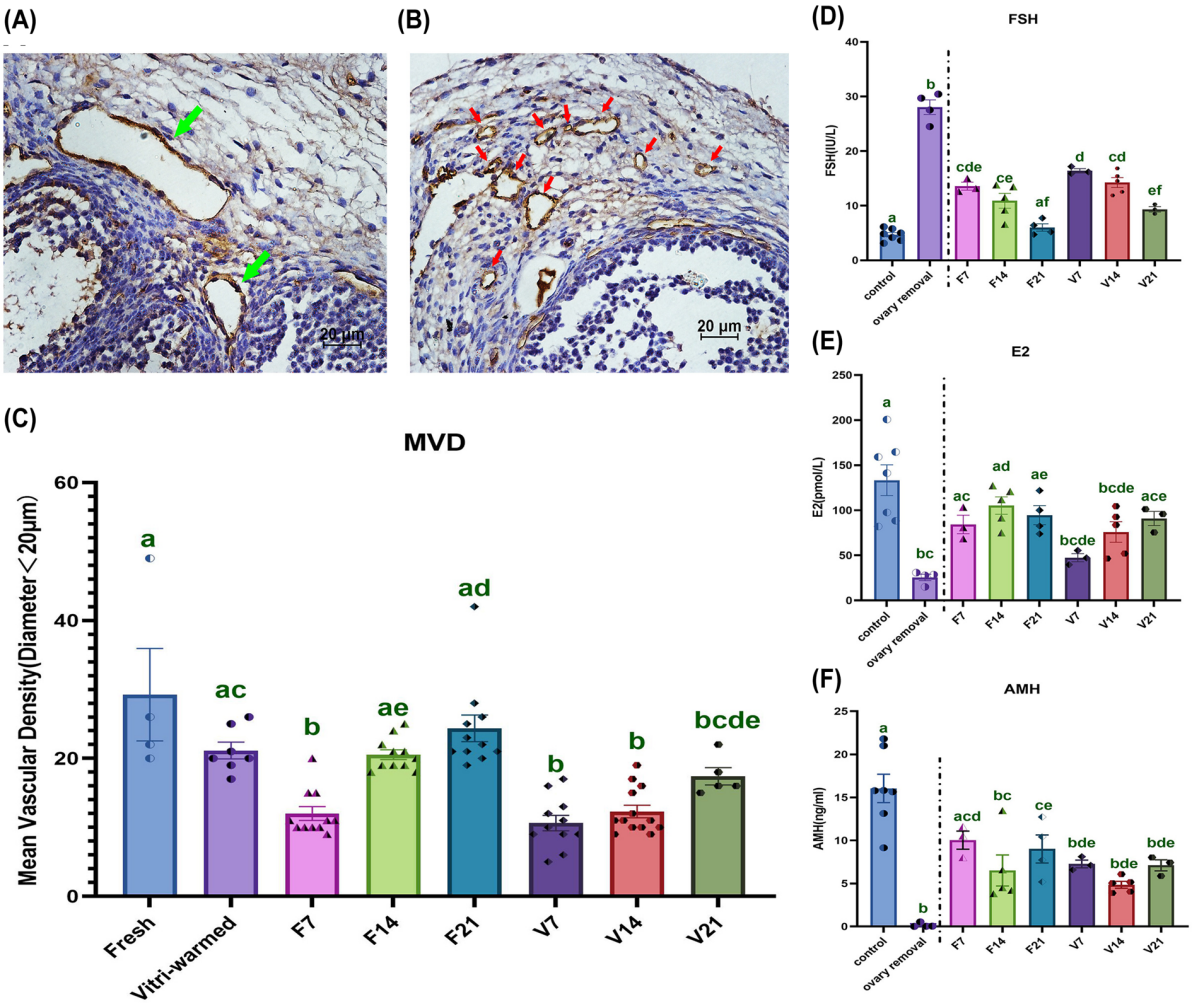
Angiogenesis in OTs and serum hormone levels (**A,B**) Immunohistochemistry for CD31. Green arrows indicate blood vessels with a diameter greater than 20 μm, red arrows indicate microvessels with a diameter less than 20 μm, and the scale bar = 20 μm. (**C**) The mean vascular density (MVD) of OTs. (**D–F**) Serum concentrations of FSH, E2, and AMH. Data are represented as means ± standard error. Individual data points are indicated by circles, triangles, and diamonds. The between group statistical significance (*P*<0.05) is indicated by lower case letters, such that the bars labelled with a certain letter are not statistically different from each other but are statistically different from the bars without that letter. The fresh ovarian transplantation groups were designated as F7, F14, and F21, with collections on post-transplantation day 7, 14, and 21, respectively. The vitri-warmed ovarian groups were similarly named V7, V14, and V21. AMH, anti-Müllerian hormone; E2, estradiol; FSH, follicle-stimulating hormone; OT, ovarian tissue.

We only counted microvessels with a diameter <20 µm because these are more representative of capillary blood perfusion in the OT (Figure 3A,B). The mean vascular density (MVD) in the grafts of both the F7 and V7 groups decreased (Figure 3C, *P*<0.05). The MVD of the fresh group gradually recovered to the same vascular condition in F21 group as that of the control group, whereas the MVD of the V21 group did not recover to the same level as that of the control group, although it did increase with time (Figure 3C).

The endocrine function of the grafts is illustrated in Figure 3D–F. Compared with the ovary removal group, the hormone levels of FSH, E2, and AMH partially recovered in both the F7 and V7 groups and showed further improvements with time. Similar to the MVD characteristics, the endocrine function of the fresh group showed a greater improvement than that of the vitri-warmed group.

### Mitochondrial function of oocytes isolated from OTs

**Figure 4:**
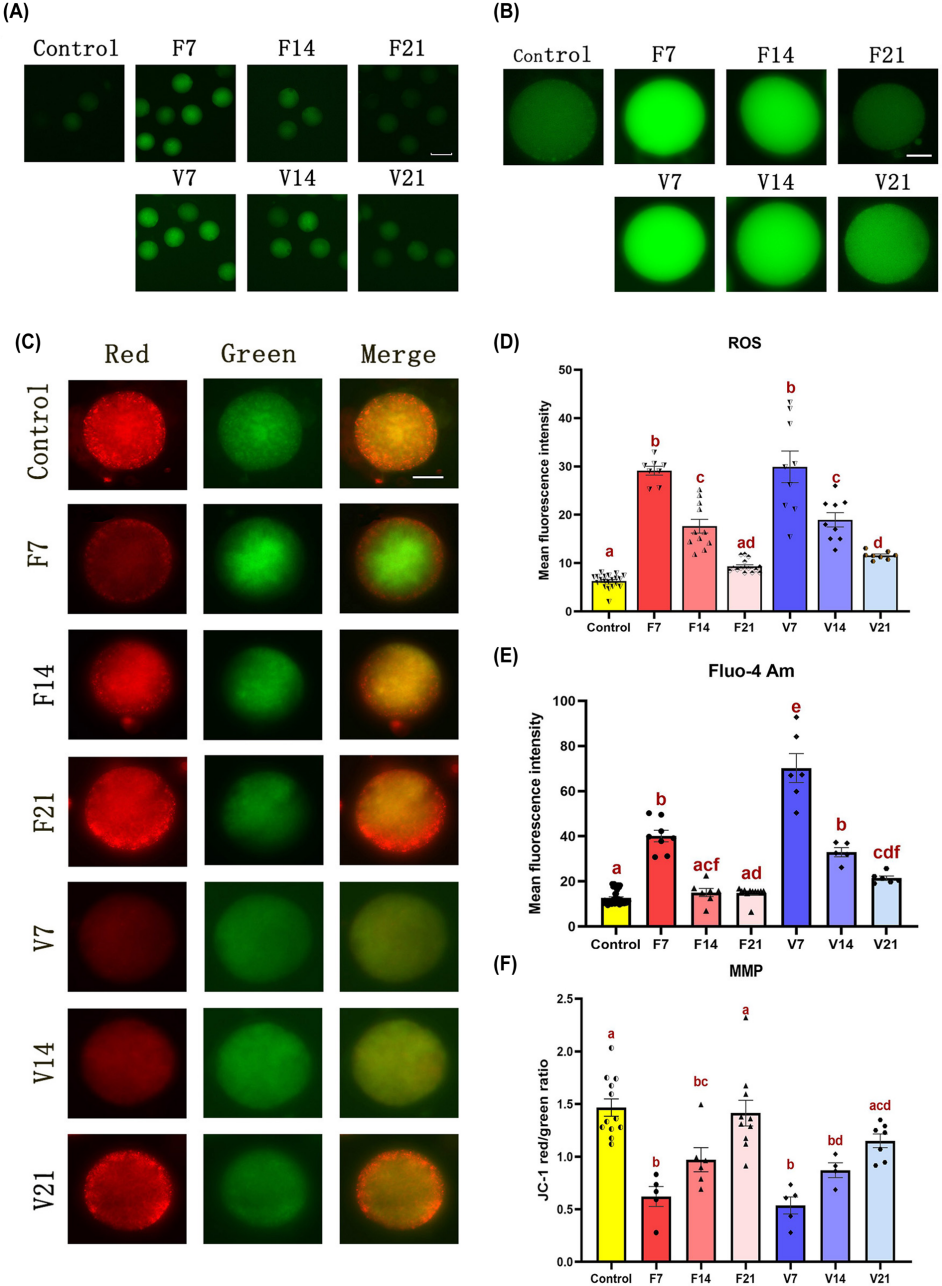
Intracellular ROS, Ca^2+^, and MMP levels detected in oocytes (**A–C**) ROS, Ca^2+^, MMP staining of oocytes; scale bar = 75 μm in (A) and 25 μm in (B and C). (**D,E**) Mean fluorescence intensity of ROS and Ca^2+^ staining of oocytes (**F**) JC-1 red/green ratio of oocytes. Data are represented as the means ± standard error. Individual data points are indicated by circles, triangles, and diamonds. The between group statistical significance (*P*<0.05) is indicated by lower case letters, such that the bars labelled with a certain letter are not statistically different from each other but are statistically different from the bars without that letter. The fresh ovarian transplantation groups were designated as F7, F14, and F21, with collections on post-transplantation day 7, 14, and 21, respectively. The vitri-warmed ovarian groups were similarly named V7, V14, and V21; MMP, mitochondrial membrane potential; ROS, reactive oxygen species.

Compared with the control group, the ROS and Ca^2+^ levels were higher in both F7 and V7 group ovaries (*P*<0.05), but gradually decreased with time (Figure 4A,B,D,E). Upon exposure to JC-1, normally functioning mitochondria stain red and stressed mitochondria stain green; as such, the red/green ratio is often used to measure MMP. Compared with the control group, the oocyte MMP levels of both the F7 and V7 group ovaries were reduced (*P*<0.05), but gradually increased with time (Figure 4C,F). By post-transplantation day 21, the MMP levels in both groups had returned to that of normal oocytes.

**Figure 5:**
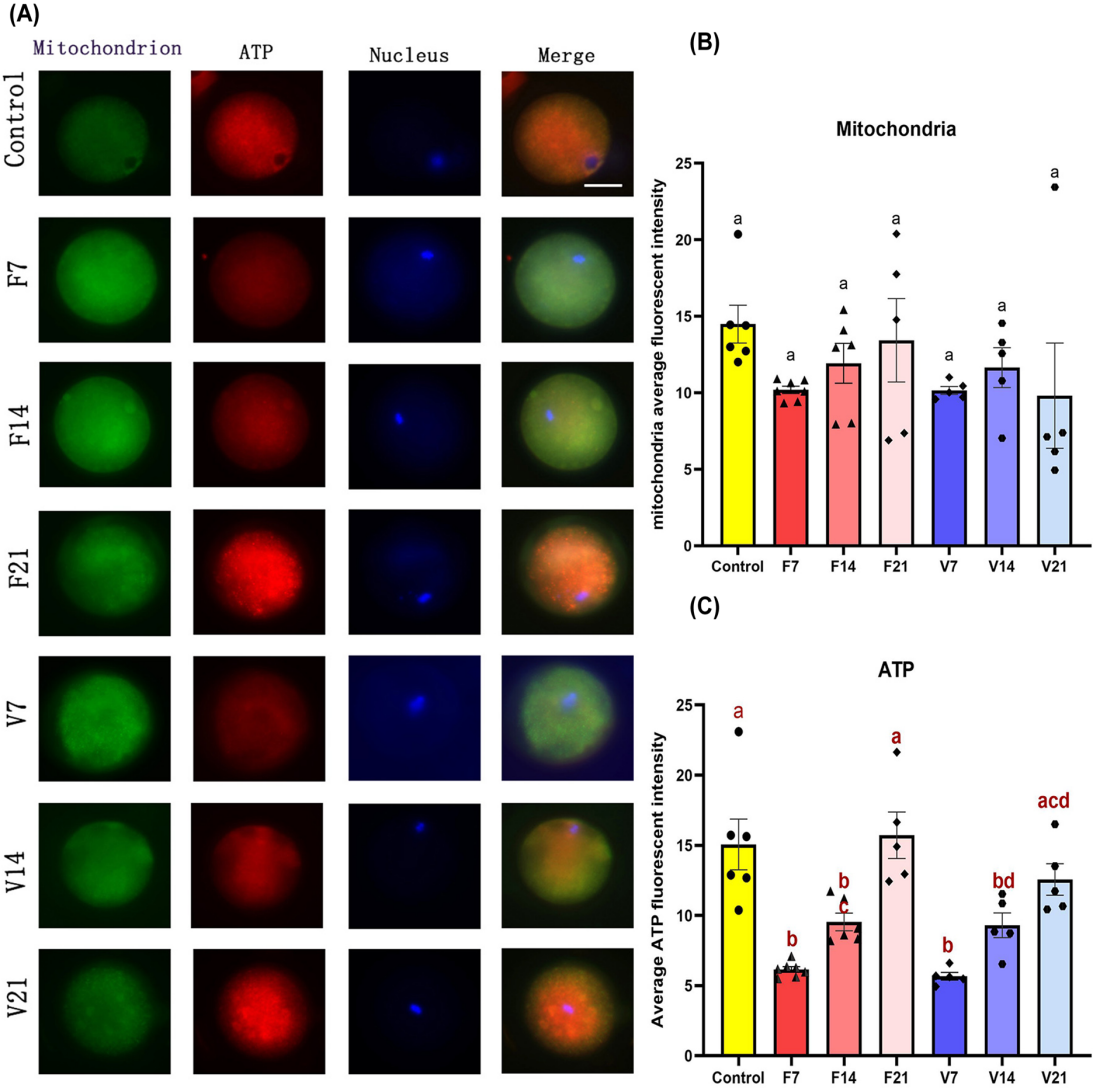
Mitochondria and ATP in oocytes (**A**) Co-staining of mitochondria and ATP using fluorescence probes. Green, red, and blue signals represent mitochondria, ATP, and nuclei, respectively; scale bar = 25 μm. (**B,C**) Mean fluorescence intensity of mitochondria and ATP staining of oocytes. Data are represented as means ± standard error. Individual data points are indicated by circles, triangles, and diamonds. The between group statistical significance (*P*<0.05) is indicated by lower case letters, such that the bars labelled with a certain letter are not statistically different from each other but are statistically different from the bars without that letter. The fresh ovarian transplantation groups were designated as F7, F14, and F21, with collections on post-transplantation day 7, 14, and 21, respectively. The vitri-warmed ovarian groups were similarly named V7, V14, and V21.

Although there was no significant difference in the number of mitochondria between groups (Figure 5B), ATP, the product of mitochondrial function, was reduced on post-transplantation day 7 (Figure 5C, *P*<0.05). This indicates that a subset of the mitochondria was non-functional early post-transplantation. Similar to the decreasing trends in ROS and Ca^2+^ levels, ATP production gradually increased with time following transplantation. On the 21st day after transplantation, the concentration of ATP in the control group was similar to that in the F21 and V21 groups (*P*>0.05).

### DNA damage detection on oocytes

**Figure 6:**
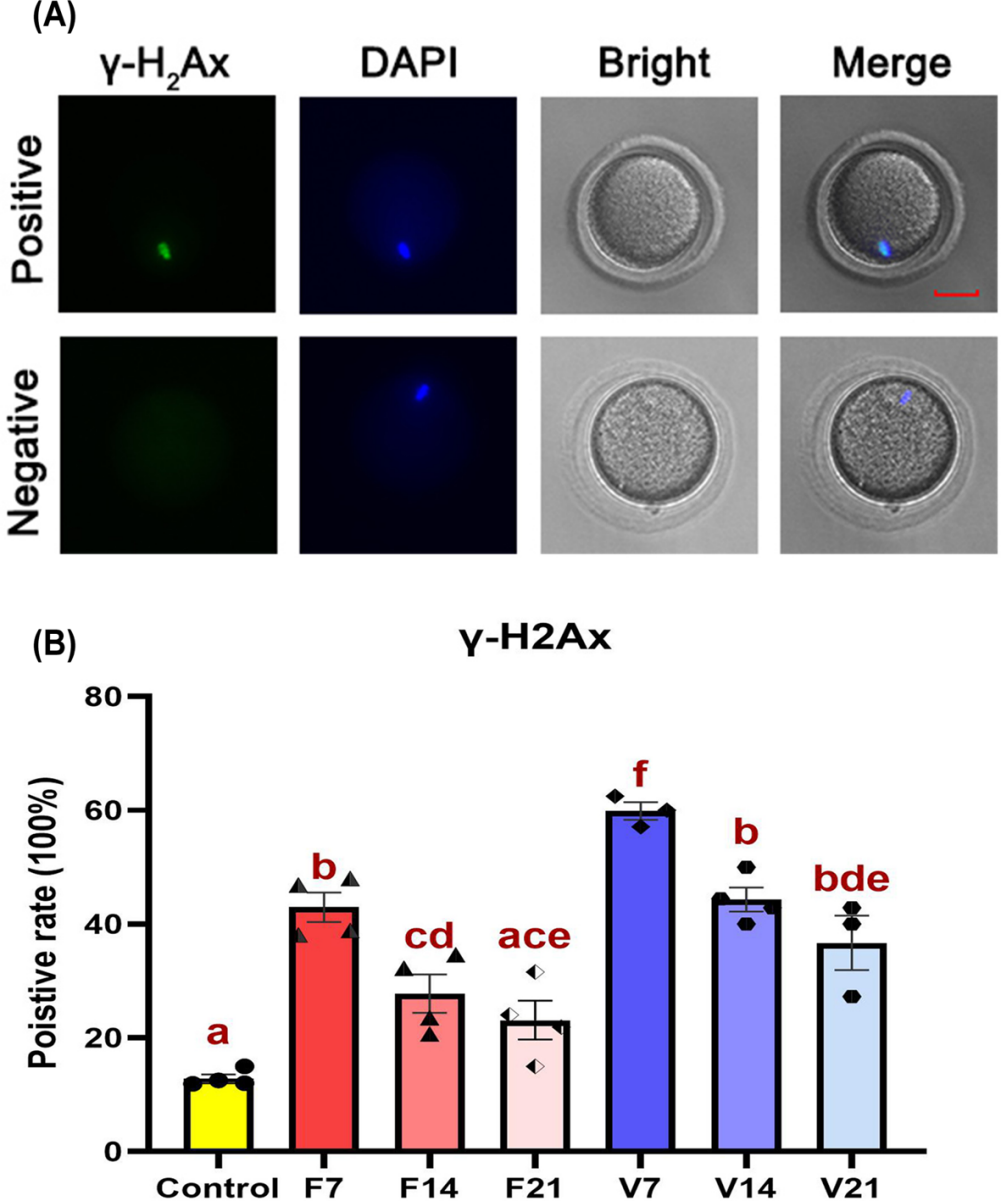
γ-H2AX protein levels in oocytes (**A**) Positive and negative expression of γ-H2AX in oocytes. The green and blue signals represent γ-H2AX proteins and oocyte nuclei, respectively; scale bar: 25 μm. (**B**) γ-H2AX positivity in oocytes. Data are represented as means ± standard error. Individual data points are indicated by circles, triangles, and diamonds. The between group statistical significance (*P*<0.05) is indicated by lower case letters, such that the bars labelled with a certain letter are not statistically different from each other but are statistically different from the bars without that letter. The fresh ovarian transplantation groups were designated as F7, F14, and F21, with collections on post-transplantation day 7, 14, and 21, respectively. The vitri-warmed ovarian groups were similarly named V7, V14, and V21; γ-H2AX, nuclear γ-H2A histone family member X.

Examination of expression of the DNA damage marker γ-H2Ax (Figure 6) showed that, compared with the control group, the percentage of γ-H2Ax-positive cells in the F7 and V7 groups increased (*P*<0.05), with the increase in the V7 group being more pronounced. However, γ-H2Ax expression gradually decreased over time post-transplantation. By the 21st day post-transplantation, the γ-H2Ax level in F21 group oocytes was similar to that in the control group; however, although the V21 γ-H2Ax level was not significantly different from that in F21 group oocytes, it was still higher than that in control oocytes (*P*<0.05).

### Embryonic development after IVF

**Figure 7:**
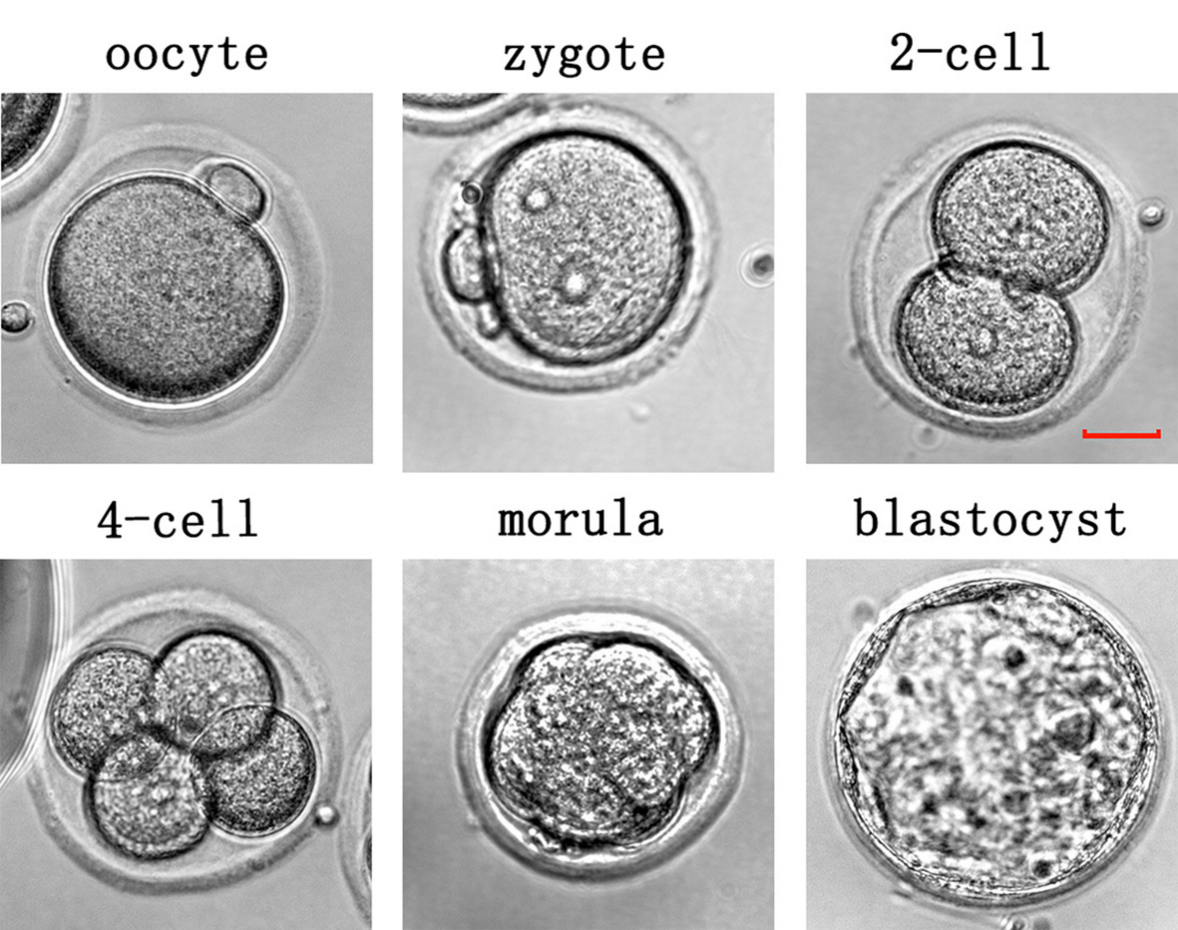
Oocyte and embryonic development at different stages following *in vitro* fertilisation; scale bar: 25 μm

Regardless of whether oocytes were from fresh or vitri-warmed transplants, after IVF, the oocytes obtained by ovulation induction on the 7th day post-transplantation were prone to blockage at the 2-cell stage (Figure 7 and Table 1). Although this phenomenon was overcome in the F14 and V14 groups, blastocyst formation remained reduced (*P*<0.05). On post-transplantation day 21, we observed no significant difference in blastocyst formation between F21 and control groups. However, although V21 was not significantly different from F21, blastocyst formation was lower than that in the control group (*P*<0.05).

**Table 1:** Embryonic formation rates in different group

	Control	F7	F14	F21	V7	V14	V21
**Number of oocytes**	243	39	72	93	31	34	30
**2-cell rate/%**	87.63 ± 1.924^*^	74.02 ± 2.432^†^	80.12 ± 3.840^*†^	86.80 ± 3.439^*^	51.85 ± 7.256^‡^	62.20 ± 5.181^‡^	73.75 ± 7.246^†^
**4-cell rate/%**	77.97 ± 3.854^*^	51.16 ± 8.893^†‡^	53.47 ± 4.083^†‡^	75.49 ± 4.479*	29.36 ± 12.21^§║^	42.66 ± 6.931^†║^	63.96 ± 3,291*^‡^
**blastocyst rate/%**	65.68 ± 3.044^*^	33.30 ± 4.312^†‡^	47.28 ± 2.607^†§^	63.28 ± 2.257^*║^	18.52 ± 6.655^¶^	32.14 ± 2.380^‡^^¶^	51.46 ± 13.12^§║^

Different superscript letters in the same row represent significant differences; *P*<0.05.

## Discussion

OTCT has gradually reached the clinical arena worldwide as an effective method for preserving female fertility. However, its efficacy is fairly low due to cryoinjury during OT cryopreservation and ischaemic injury after OT transplantation [6,7]. To improve the efficiency of OTCT, many researchers have recently performed studies on methods to improve the success rate of OTCT and reduce the loss of follicles in OT by adding antioxidants or angiogenesis factors to the culture medium [15–19]. We believe that in addition to the loss of the number of follicles, the quality of follicles also warrants attention. At present, most reproductive centers recommend initiating assisted reproductive treatment at 4-6 menstrual cycles after transplantation [21]. However, some doctors may consider performing early assisted reproductive treatment as the life span of transplanted ovarian slices is significantly shorter than that of normal ovaries [21], or patients may require early assisted reproductive treatment for other personal reasons after achieving cancer cure. Herein, we examined the quality of ovaries and oocytes within approximately 5 murine menstrual cycles (21 days) after transplantation, with the aim of investigating the effects of ischaemic injury and/or cryoinjury on ovarian and oocyte quality during the early stage.

In the present study, we found that MVD decreased in the early post-transplantation period but increased gradually with time, which was consistent with the previously reported results of 3D printing of ovarian microvessels post-transplantation [22], suggesting that the OT is in a hypoxic-ischaemic environment at this point. However, Lee et al. found that the increase in CD31 staining in OT was not clearly observed on post-transplantation day 7 [7], possibly owing to differences in statistical methods, wherein only the number of CD31-positive areas was counted on different days after transplantation; in contrast, the number of CD31-positive microvessels was counted in the present study to estimate OT microcirculation.

We observed a decrease in both follicle numbers via HE staining (Figure 2C) and AMH post-OTCT. AMH is generated in the preantral and antral follicles of ovaries by surrounding granulosa cells and is therefore considered a marker of ovarian reserve [23]. The AMH decline post-OTCT generally represents a decrease in follicle number [24]. AMH reduction in the vitri-warmed group was greater than that in the fresh group and still lower than that in the control group until post-transplantation day 21, indicating that this OT follicular reduction was the combined effect of cryoinjury and ischaemic injury. Furthermore, our finding that changes in FSH, a biomarker of ovarian response to adenohypophysis [25], are consistent with those in AMH is indicative of the gradual recovery of transplanted ovaries over time and that the vitri-warmed group experienced greater difficulty in recovery. E2 monitoring is considered useful for assessing follicular growth [23] because its elevation post-transplantation represents the activation of follicular growth. FSH/E2/AMH levels reflected the trends in follicular reserve, ovarian reactivity, and endocrine function post-OTCT. In addition, HE staining of the F7 and V7 groups revealed a large number of antral follicles. This may be related to the accumulation of large quantities of ROS in OT, leading to abnormal follicular activity [12,26]. Although many follicles are activated, our subsequent assays showed that these follicles have various energy metabolism abnormalities and are not suitable for fertilisation, indicating the wasting of limited ovarian reserves.

To extract oocytes from OT at an appropriate time, we performed controlled ovarian hyperstimulation in mice. Our previous study showed that oocyte quality produced by injecting 10 IU of PMSG into mice did not differ from that of normal oocytes; however, if the PMSG dose exceeds 10 IU, a series of energy metabolism abnormalities is triggered in oocytes, resulting in poor embryonic development after IVF [27]. Interestingly, in this experiment, we inadvertently found that in mice undergoing ovarian transplantation, when the dose of PMSG reached 20 IU in the early post-transplant period, the produced oocytes still showed excellent energy metabolism and embryonic development, possibly owing to the insufficient perfusion of transplanted OT blood vessels; thus, the amount of drug absorption was limited, or the transplanted OTs did not respond well to ovulation-stimulating drugs. This hypothesis is consistent with the MVD and FSH results shown in Figure 3. Although we did not investigate this phenomenon further as it was beyond the scope of the present study, we hope that this finding will nevertheless be useful to clinicians when they contemplate gonadotropin dosages for patients undergoing ovulation induction post-OTCT.

After isolating oocytes from OTs, we characterised the mitochondrial function in oocytes that grew and matured in a hypoxic-ischaemic environment. The oocyte energy metabolism status on different days post-transplantation was consistent with the trend of MVD. ROS levels were significantly increased in oocytes at the early stage of ovulation post-transplantation, and ROS accumulation can cause abnormal energy metabolism in oocytes [28,29]. Under normal circumstances, the production and removal of ROS maintain a dynamic balance during oocyte development and maturation [28,30]. However, excessive ROS levels can lead to oxidative stress and affect mitochondrial function, thereby reducing ATP synthesis and affecting meiotic spindle assembly in oocytes [31]. When some mitochondria are non-functional in the early stages post-transplantation, the amount of intracellular ATP decreases, and the endoplasmic reticulum is stimulated to release a large amount of Ca^2+^. This Ca^2+^ wave enters the mitochondrial matrix to accelerate tricarboxylic acid circulation and promote the production of ATP in the respiratory chain, reducing the intracellular energy deficit [32,33]. Conversely, MMP levels on oocytes determine mitochondrial functions, including ATP production and Ca^2+^ homeostasis. When MMP levels decrease, mitochondrial swelling, ATP hydrolysis, and Ca^2+^ homeostasis is abolished, which in turn affects embryonic development [34]. In addition to energy metabolism disorders caused by ischemic injury, several studies have shown that the freezing process results in hypothermia-hypoxia, which triggers a decrease in ATP levels and is linked to the decoupling of the internal mitochondrial membrane. This produces an elevation in oxygen levels, as well as an increase in ROS and cell death [35]. Overall, our study found that oocytes isolated from early ischaemic OTs post-transplantation often had abnormally elevated intracellular ROS and calcium concentrations compared with those in the control group, while ATP and MMP levels decreased. This phenomenon triggers chromosomal aberrations in oocytes and reduces embryonic developmental competence, thereby reducing the success rate of assisted reproductive technology [31,36].

γ-H2Ax is an early indicator of DNA damage, and its expression was increased in oocytes at the early stage post-transplantation, indicating that ROS accumulation in oocytes causes DNA double-strand breaks [37]. Moreover, mitochondrial dysfunction and DNA damage directly affect fertilisation and embryonic development. If the mouse embryo undergoes oxidative damage, a 2-cell stage block post-fertilisation will be triggered due to zygotic genome activation [38–40]. If oxidative damage is not repaired once the 2-cell block is overcome, blastocyst formation is inhibited [41,42]. Overall, the results of the present study demonstrated that a 2-cell block occurred in oocytes produced from OTs on post-transplantation day 7, while a decline in blastocyst formation occurred in oocytes produced by ovaries on post-transplantation day 14. Compared with the control group, the fresh group showed no significant difference in embryonic development on day 21 post-transplantation, whereas the vitri-warmed group still demonstrated a decline in blastocyst formation when the vasculature was stabilised by day 21 [12]. Thus, the developmental potential of oocytes isolated from OT can be considered to be related to the number of days after transplantation.

Overall, we found no significant differences in either ovaries or oocytes between the control and fresh groups or between the fresh and vitri-warmed groups after day 21 post-transplantation. This suggests that cryoinjury does not occupy a dominant position in OTCT, as shown in previous research [7]. However, when cryoinjury and ischaemic injury coexist, a synergistic effect may weaken ovary and oocyte function post-OTCT. Despite this trauma, oocyte quality improved with increasing time post-transplantation owing to the gradual increase in vascular networks. As such, despite an increase in injuries post-transplantation, OTCT remains a feasible fertility preservation method. However, pregnancy and live birth rates remain poor in patients who have undergone OTCT compared with those who have conceived naturally [6,43], and many studies have confirmed that one reason for this is the follicular loss in OTCT [7,44], therefore, they focused on follicle loss reduction. However, we also believe that poor oocyte quality is an important factor that must be taken into consideration. These injuries in oocytes usually lead to spindle instability, chromosomal abnormalities, and telomere shortening [31,36]. However, whether this damage will gradually be repaired during embryonic development after fertilisation remains unclear. Further, if the damage is not repaired, the potential health effects in the resulting offspring are unknown. Our future research goal is to study the health of offspring produced by OTCT. Of course, despite mice are commonly used animal models for human reproductive medicine research, it is unclear how far these results can be extrapolated from mice to the human ovary. Overall, our results suggest that ovarian cryopreservation is suitable for fertility preservation in prepubertal girls and women who cannot postpone treatment before chemoradiotherapy. Due to the ability to preserve ovarian ovulation and endocrine function, this technique is invaluable for patients with endometriosis, early premature ovarian insufficiency, and menopausal women who need to maintain endocrine function, and thus has broad clinical application prospects in the near future.

## Clinical perspectives

- In order to help clinicians understand the benefits and shortcomings of OTCT, we used a murine model to characterise the OT and oocytes produced within 21 days after ovarian transplantation.
- The results showed that cryoinjury and ischaemic injury during OTCT can cause a reduction in oocyte numbers in the grafts, dysfunctional mitochondria, and oocytes DNA damage, which may adversely affect embryonic development. Oocyte loss post-OTCT is irreversible, but oocyte damage is gradually repaired post-transplantation.
- Overall, our results suggest that ovulation induction and assisted pregnancy treatment should not be performed too early after OT transfer. Otherwise, the abnormal energy metabolism of the oocytes may pose a health hazard to the offspring. It should be noted that the mouse model may not be directly applicable to humans. As such, the decision regarding whether to start assisted reproductive treatment early after ovarian transplantation should be made following comprehensive consideration of various factors.

## Data Availability

The data underlying this article are available from the corresponding author upon reasonable request.

## Abbreviations

γ-H2AX: nuclear γ-H2A histone family member X
AMH: anti-Müllerian hormone
E2: estradiol
HCG: human chorionic gonadotropin
FSH: follicle-stimulating hormone
MMP: mitochondrial membrane potential
MVD: mean vascular density
OT: ovarian tissue
OTCT: ovarian tissue cryopreservation and transplantation
PMSG: pregnant mare serum gonadotropin
ROS: reactive oxygen species
